# Whey Protein Lycosome Formulation Improves Vascular Functions and Plasma Lipids with Reduction of Markers of Inflammation and Oxidative Stress in Prehypertension

**DOI:** 10.1100/2012/269476

**Published:** 2012-12-24

**Authors:** Ivan M. Petyaev, Pavel Y. Dovgalevsky, Victor A. Klochkov, Natalya E. Chalyk, Nigel Kyle

**Affiliations:** ^1^Lycotec Ltd., Granta Park Campus, Cambridge CB21 6GP, UK; ^2^Institute of Cardiology, Chemyshevskogo Street, Saratov 410028, Russia

## Abstract

Parameters reflecting cardiovascular health and inflammation were studied in a pilot clinical trial conducted on 40 patients with prehypertension. The patients were treated with a new proprietary formulation of a whey protein (WP) isolate embedded into lycopene micelles (WPL) during a 1-month period. Control groups received lycopene or WP as a singular formulation or placebo pills for the same period of time. Combined WPL formulation of whey protein and lycopene has caused multiple favorable changes in the cardiovascular function (including a tendency to the reduced systemic blood pressure), the plasma lipid profile, and the inflammatory status of patients with prehypertension, whereas singular formulations of the compounds and placebo did not have such an effect. The reduction of plasma triglycerides and cholesterol fractions and almost two-fold decline in C-reactive protein (CRP) and inflammatory oxidative damage (IOD) levels as well as an increase in nitric oxide (NO), tissue oxygenation (StO_2_), and flow-mediated dilation values constitute the most significant benefit/outcome of the treatment with the combined formulation of whey protein and lycopene. The treatment did not affect the values of ankle-brachial index (ABI), body weight, and body mass index (BMI).

## 1. Introduction

Whey proteins and whey-related peptides represent a newly emerging class of biological substances with as yet poorly understood potential benefits for human health. For decades whey has been considered as a fairly useless liquid byproduct from the production of cheese. However, modern methods for the fractionation of whey allow the isolation of a variety of substances with an enormously wide spectrum of biological activity [1]. Undenatured whey proteins have been shown to ameliorate multiple metabolic abnormalities under both experimental and clinical conditions. Whey protein has recently been reported to have a significant beneficial impact on the nonalcoholic fatty liver disease, plasma lipid profile, and the suppression of oxidative stress in rats [2–4] as well as glucose tolerance and insulin sensitivity in mice fed with a high-fat diet [5]. Similar observations have been made in a clinical setting. Reduced intrahepatic lipid accumulation in obese female patients [6] and amelioration of macrovesicular steatosis in NASH [7], elimination of elevated plasma triglyceride and cholesterol levels in hypercholesterolemia [8], reduced LDL [9] as well as improved insulin sensitivity in insulin-resistant individuals [10], increase in antioxidant capacity [7], broad antibacterial activity [11, 12], and inhibition of inflammation including in cardio- and cerebrovascular patients [9, 13] have all been attributed to whey protein constituent intake (lactoferrin, immunoglobulins, glutamine, lactalbumin, and their peptides) in interventional clinical trials. 

Moreover, there is a growing number of whey-derived peptides whose pharmacological activity *in vitro* systems is comparable to that of well-known pharmacological agents. As an example, three newly identified peptides derived from  *α*-lactalbumin (AA 16–26, KGYGGVSLPEW),  *α*-lactalbumin (AA 97–104, DKVGINYW), and *β*-lactoglobulin (AA 33–42, DAQSAPLRVY) have been shown to have a remarkable inhibitory effect against angiotensin-converting enzyme, ACE [14]. In another publication, five more antihypertensive peptides, lactokinins, were identified [15]. The most potent lactokinin to date has an ACE IC50 of 42.6 *μ*mol [15].

Despite their high inhibitory potency, whey-derived proteins and peptides are highly susceptible to gastrointestinal enzymes which undermine their oral use in treatment of hypertension. This is the main reason why most clinical trials on whey proteins or their derivatives require a significant quantity to be consumed daily. Indeed, susceptibility of whey protein and its derivates to the intestinal enzymes is a major problem complicating their therapeutic use. It explains variations in individual responses to whey protein administration among patients and dictates the necessity for the development of new whey protein formulations development providing a degree of protection for the whey protein and its peptides from the intestinal environment. This would consequently increase their bioavailability and, as result, of this their efficacy.

In the present paper, we report the results of a pilot clinical trial with a new proprietary formulation of a whey protein (WP) isolate embedded into lycopene micelles [16]. Lycopene was chosen as a protector because there are no known digestive enzymes in the human gastrointestinal tract which can break down this carotenoid. Parameters reflecting cardiovascular health and inflammation were studied in 40 patients treated with WP-lycopene proprietary formulation during a 1-month period. Control groups received lycopene or WP as a singular formulation or placebo pills for the same period of time. 

## 2. Material and Methods

### 2.1. Study Design

The study was conducted at the Institute of Cardiology, the Ministry of Health of the Russian Federation (Saratov, RF), during 2010-2011. The protocol was approved by the local ethics committee. All patients were informed about the purpose of the study and have given written consent regarding their participation in the study. 

### 2.2. Inclusion/Exclusion Criteria

The study was part of a larger multiarm trial for which 107 patients with prehypertension fulfilling clinical inclusion criteria were selected. Prehypertension was defined according to the USA Joint National Committee, JNC 7 [17], as a state with the systolic blood pressure (SPB) between 120 and 139 mm Hg and diastolic blood pressure (DBP) between 80 and 89 mm Hg. Prehypertensive patients were further screened for levels of blood markers for oxidative stress and inflammation. 86 patients were positive for both types of markers. 40 patients were finally selected for this study and were randomized into four groups of 10 patients each. 5 of the initially enrolled patients could not complete the study for reasons not related to the intake of the test products. They were replaced with eligible patients from the prescreened pool. Patient's eligibility for the study was determined by the following inclusion/exclusion criteria.

*Inclusion Criteria*

- Caucasian male or female subjects 45–73 years old.
- Signed informed consent.
- Non- or light-to-moderate smokers (≤10 cigarettes daily).
- Sustained resting systolic blood pressure 120–139 mm Hg.
- Sustained resting diastolic blood pressure 80–89 mm Hg.
- Serum markers for inflammation Chl.pn-IgG ELISA × 10^3^ ≥ 400 and CRP ≥ 6 *μ*g/mL.
- Serum markers for oxidative stress LDL-Px ELISA × 10^3^ ≥ 250 and IOD ≥ 40 *μ*M/mL.
- No antihypertensive, lipid-lowering or any other cardiovascular drugs.
- Willingness and ability to comply with the protocol for the duration of the study.

*Exclusion Criteria*

- Unwillingness to sign informed consent.
- Unable to comply with the protocol for the duration of the study.
- History of MI in the 3 months preceding the study.
- Ejection fraction (EF) < 45%.
- Significant medical condition that would impact safety considerations (e.g., significantly elevated LFT, hepatitis, severe dermatitis, uncontrolled diabetes, cancer, severe GI disease, fibromyalgia, renal failure, recent CVA (cerebrovascular accident), pancreatitis, respiratory diseases, epilepsy, etc.).
- Compulsive alcohol abuse (>10 drinks weekly), or regular exposure to other substances of abuse.
- Participation in other nutritional or pharmaceutical studies.
- Resting heart rate of >100 beats per minute or <45 beats per minute.
- Positive test for tuberculosis, HIV, or hepatitis B.
- Did not tolerate phlebotomy.
- On a special diet in the 4 weeks prior to the study (e.g., liquid, protein, raw food diet).
- Tomato or milk intolerance.

### 2.3. Products

The first product was whey protein isolate (WP), Prolacta (Lactalis, France). The daily dose was given as 1 capsule containing 70 mg of WP. The second control product was lycopene (Vitatene, Spain). The daily dose was 1 capsule containing 7 mg of lycopene. The third product was a complex of WP embedded into protective lycopene matrix, whey protein lycosome (WPL, Lycotec, UK) [16]. The daily dose of this composite was 1 capsule containing 70 mg of the WP and 7 mg of lycopene. All products were to be taken with the main evening meal. The period of administration was 1 month. 

## 3. Methods 

### 3.1. BMI, Pulse Rate, and BP

To measure body mass index, BMI, body mass of the patients, and their height were measured in the morning and BMI was calculated in kg/m^2^. Pulse rate, systolic and diastolic blood pressure, SBP, and DBP were measured three times in the left arm of the seated patient after 15 min of rest. The time between measurements was no less than 2 minutes. The mean number for each parameter was calculated.

All body and vascular parameters were measured in the morning between 8 and 10 am. 

### 3.2. Flow-Mediated Dilation (FMD)

Endothelium-dependent flow mediated vasodilatation was measured in accordance with traditional guidelines accepted in the last 10 years [18, 19]. Patients were tested under ambient conditions at the same time of morning while the patients were in a supine position. 

High resolution ultrasound was used at the same anatomical landmark of a section of the brachial artery for a period of 30 sec before and during the peak of reactive hyperaemia. It was applied prior to sphygmomanometer cuff occlusion and 1 min after its deflation. The level of inflation was 50 mm Hg above the patient's systolic blood pressure and it lasted for 5 minutes. Arterial diameter was imaged above the antecubital fossa in a longitudinal scan by duplex ultrasound with linear phase-array transducer. FMD was calculated as a change in poststimulus diameter as a percentage of the baseline diameter [20]. 

### 3.3. Ankle-Brachial Index (ABI)

Between left and right brachial arteries, the one with the highest SBP, and between left and right tibial arteries, the one with the highest SBP were chosen for the assessment of ABI. For this purpose, a continuous-wave Doppler probe was used after patients had been in a supine position for at least 15 min of rest [21, 22]. 

### 3.4. Tissue Oxygenation

As a tissue target for the assessment of oxygen saturation, StO_2_, or combined level of oxygenated haemoglobin and myoglobin, we used the the Nar eminence and forearm muscles of the patients. StO_2_ was analysed by continuous wavelength near-infrared spectroscopy, NIRS, with wide-gap second-derivative (In Spectra, Hutchinson Technology, MN, USA). The measurements were made at different time points. The recording was started after 15 min of rest in a supine position before occlusion of the brachial artery. It was then continued during stagnant ischemia induced by rapidly inflating the cuff to 50 mm Hg above systolic BP. The ischemia lasted for 3 min, and the recording period lasted for another 5 min after that until StO_2_ was stabilized [23, 24]. 

Then the area under the hyperaemic curve, AUC, of the recorded signal for the settling time in the postocclusion period was calculated as described earlier in % O_2_/minute [25, 26].

### 3.5. Blood Collection

Blood was collected in the morning after night fast from arm veins of the patients. The plasma was separated from the rest of the clotted mass by centrifugation, then aliquots were stored at −80°C prior to analysis. 

### 3.6. Biochemistry and Inflammatory Markers

Glucose, total cholesterol, TC, triglycerides, TG, high density cholesterol, HDL, low density cholesterol, LDL, C-reactive protein, CRP, *Chlamydia pneumoniae* IgG (Chl.pn-IgG), and NO_*x*_ were measured using commercially available analytical kits according to the manufacturers' recommendations (ByoSystems, Medac, R&D Systems).

### 3.7. Inflammatory Oxidative Damage (IOD)

Plasma samples were incubated overnight in 0.05 M PBS acetate buffer (pH 5.6) which would imitate the type of oxidative damage which occurs during the release of lycosomes following neutrophil degranulation. The following morning, the reaction was terminated using trichloroacetic acid. The concentration of the end products such as malonic dialdehyde (MDA), and other possible thiobarbituric acid reactive substances, TBARS, was then measured by colorimetric methods [26] using reagents and kits from Cayman Chemical (MC, USA). 

### 3.8. LDL-Px

Activity of serum LDL peroxidase proteins, which include IgG with superoxide dismutase activity [27], was measured as described earlier [28, 29]. 

### 3.9. Statistics

For the assessment of normally distributed parameters, the Shapiro-Wilk method was used. Student's  *t*-test was then applied both for paired and unpaired samples. In cases where parameters were not normally distributed Mann-Whitney  *U*  test and Kruskal-Wallis test were used. ANOVA and ANCOVA were used with post hoc analysis (Statistica 9 suit, StatSoft; Inc.). Statistical significance between two-tailed parameters was considered to be  *P* < 0.05.

## 4. Results

Table 1 summarizes the background information of patients enrolled in the clinical trial. Similarities in gender, age, and BMI parameters between different groups suggest effective randomization and internal validity of the four major groups comprising the study. All of the patients remained in the normoglycemic range at the enrolment stage and at the end point of the study.

### 4.1. Changes in Lipid Profile

As can be seen from Table 2, neither treatment with singular formulations of WP or lycopene was accompanied by changes in plasma lipid profile. In contrast, the combined formulation (WPL) caused a statistically significant reduction in total cholesterol and LDL cholesterol. The most significant changes were seen in the plasma triglyceride level (reduced by 34.1% as compared to the control value). Treatment with WPL was also associated with some increase in HDL cholesterol. No changes in plasma lipid profile were seen in the placebo group. 

### 4.2. Markers of Oxidative Stress, Inflammation, and NO_*x*_

As shown in Table 3, treatment with singular formulations of WP and Lycopene did not affect the markers of oxidative damage and inflammation studied. Only IOD values were decreased in the lycopene-treated patients. Nevertheless, the WPL combinatory treatment led to a statistically significant decline in plasma concentration of CRP, LDL-Px, and IOD. This decline was also accompanied by a reduction in *C. pneumoniae*-specific IgG. In addition, there was a measurable increase in plasma NO_*x*_ levels validated by statistical analysis in patients treated with the combined formulation of WP and lycopene. No changes in the parameters of inflammation and oxidative stress were seen in the placebo group. 

### 4.3. Cardiovascular Parameters

None of the treatments affected values for pulse rate and ABI (Table 4). However, the combinatory formulation of WP and lycopene caused a statistically significant increase in flow-mediated dilation and StO_2_ values. The latter increase exceeded control level by 43.8%. Moreover, treatment with WP embedded into lycopene gave some decrease in the values of systemic blood pressure as revealed by statistical trend analysis. The monoformulations and placebo treatment did not show significant changes in the cardiovascular parameters.

## 5. Conclusions

Prehypertension is a progressive human disease which often develops without any specific identifiable cause and involves multiple functional changes in microcirculation, inflammatory response, and plasma lipid profile [30]. Although the precise nature of etiological factors implemented in the pathogenesis of prehypertension has yet to be identified, it is broadly assumed that early changes in lifestyle along with timely pharmacological intervention can reverse or attenuate the progress of the disease. Multiple sources of data from prospective observational, cohort, and randomized controlled clinical trials have shown that hypertension can be effectively managed by diets, food, and certain nutrients. These results create a rationale for forthcoming nutritional trials to unveil the potential use of nutrients in the management and treatment of hypertension. Since hypertension treatment requires chronic and perhaps life-long intervention, a pharmaceutical approach here would be highly challenging due to the possible risk factors and uncertainty of administering a “foreign” drug compound. A more appropriate and safer option would be the use of compounds or substances of food origin with established activity. They could be consumed in the form of a nutraceutical, fortified food or beverage. 

The major finding of the clinical trial reported above is that a combined WPL formulation of whey protein and lycopene causes multiple favorable changes in cardiovascular function (including tendency to the reduction of the systemic blood pressure), plasma lipid profile, and inflammatory status of patients with prehypertension, whereas singular formulations of the compounds do not have such an effect. The reduction of plasma triglycerides and cholesterol fractions and almost two-fold decline in CRP and IOD levels as well as an increase in NO_*x*_, StO_2_ and flow-mediated dilation values constitute the most significant benefit/outcome of treatment with the combined formulation of whey protein and lycopene. Pleiotropy as well as synergetic and antagonistic interactions between different nutrients represents keystone features governing the physiological response to dietary interventions. Therefore, any given mixture of nutrients may lead to a physiological response not independently obtainable from each individual constituent. Indeed, multiple favorable changes in the cardiovascular system achieved by treatment with a combination of WP and lycopene were not seen in our study in the groups of patients treated with WP or lycopene alone. It is worth mentioning that WP derivates and lycopene have remarkably distinctive patterns of physiological and pharmacological activity. Multiple *in vitro* and *in vivo* studies indicate that WP derivates have various effects on microcirculation, vascular tone and blood pressure [1–14] as opposed to the predominant and well-known effects of lycopene on biological oxidation, cholesterol synthesis, and inflammation. However, the full benefit of these health protecting properties is hardly traceable in the whole human body. In the case of WP, it is a privilege of infants fed with their mother's milk. With age, the gastrointestinal tract starts to produce a powerful protein digestive system of stomach pepsin and other factors. Therefore, consumption of any dairy products by adults results in almost complete breakdown of protein to peptides and even to amino acid level. In this paper, we present results which illustrate that using other nutritional molecules such as high carotenoids, and lycopene in particular, which are not significantly modified in the stomach or intestine lumen, it can provide some level of protection to a whey protein product. As result of this, its component(s) could display their intrinsic anti-inflammatory activity. We cannot claim that either protein or peptide molecules themselves reached the liver (and beyond) and acted there or that they reached “only” the small intestine and suppressed inflammation there. Whatever the locus or tissue of action, whether it is one or multiple, inhibiting one step in the cascade may suppress activity in other related tissues.

These changes in inflammatory activity were accompanied by a reduction in the level of oxidative stress. We cannot claim that they were the consequence of anti-inflammatory activity of WPL or that the whey protein that isolates itself contained antioxidant components which were able to take effect when they were delivered in the lycosome format. 

Level of NO production, flow mediated dilation, and tissue oxygenation are inversely correlated to markers of inflammation and oxidative stress. Therefore, we again cannot be certain here whether the improvement of these parameters is a result of anti-inflammatory and anti-oxidant activity of WPL or a result of intrinsic vasoactive molecules in the protein isolates itself. Or perhaps it was a combination of both possibilities.

In conclusion, it is possible to say that the improved protection of whey protein product by lycosome could allow people to improve their metabolic parameters, vascular function, and antiageing microcirculation. Perhaps this resembles the similar practice of embedding cheese and whey products into lycopene-rich tomato sauce which is one of the factors behind the longevity and cardiovascular health provided by the Mediterranean diet.

Although additional studies are required to confirm and extend our results, the results presented above represent a significant step forward in the management of hypertension and cardiovascular disease. 

## Figures and Tables

**Table 1:** Clinical groups.

Parameters	Patient group			
	WP	Lycopene	WPL	Placebo
Number of patients	10	10	10	10
Male/female	6/4	4/6	5/5	5/5
Age	57.8 ± 3.5	52.1 ± 5.6	58.4 ± 3.2	51.1 ± 5.2
Light or moderate smokers	2	1	2	2
Body mass index, in kg/m^2^	25.9 ± 2.8	28.3 ± 3.1	27.2 ± 3.4	26.8 ± 5.7
Fasting glucose, mg/dL	85 ± 4.5	82 ± 3.9	67 ± 6.8	79 ± 5.2

**Table 2:** Changes in lipid profile in prehypertensive patients treated with whey protein and lycopene formulations.

Product	TC	TG	LDL	HDL
	in mg/dL	in mg/dL	in mg/dL	in mg/dL
WP baseline after 1 month (*n* = 10)	215 ± 9.9	147 ± 10.1	133 ± 13.2	40.7 ± 0.6
	222 ± 11.3	151 ± 12.3	132 ± 11.5	40.9 ± 0.7
	*P* > 0.05	*P* > 0.05	*P* > 0.05	*P* > 0.05

Lycopene baseline after 1 month (*n* = 10)	199 ± 10.2	145 ± 9.4	142 ± 9.9	38.5 ± 0.7
	191 ± 9.8	139 ± 10.1	141 ± 8.7	39.1 ± 0.6
	*P* > 0.05	*P* > 0.05	*P* > 0.05	*P* > 0.05

WPL baseline after 1 month (*n* = 10)	209 ± 9.1	148 ± 14.5	159 ± 11.0	39.6 ± 1.2
	180 ± 8.0	97.5 ± 7.5	132 ± 7.8	44.5 ± 1.1
	*P* < 0.05*	*P* < 0.01*	*P* < 0.05*	*P* < 0.05*

Placebo baseline after 1 month (*n* = 10)	185 ± 10.3	135 ± 11.2	146 ± 12.4	40.1 ± 1.0
	182 ± 9.6	141 ± 10.5	144 ± 11.7	40.6 ± 0.9
	*P* > 0.05	*P* > 0.05	*P* > 0.05	*P* > 0.05

**Table 3:** Effect of markers of oxidative stress, inflammation, and NO.

Product	Chl.pn-IgG	CRP	LDL-Px	IOD	NO_*x*_
	ELISA × 10^3^	in *μ*g/mL	ELISA × 10^3^	in *μ*M	in *μ*M/L
WP baseline after 1 month (*n* = 10)	639 ± 55	8.0 ± 1.0	482 ± 53	97 ± 11	31 ± 6.3
	664 ± 62	7.4 ± 0.6	467 ± 62	94 ± 10	29 ± 5.5
	*P* > 0.05	*P* > 0.05	*P* > 0.05	*P* > 0.05	*P* > 0.05

Lycopene baseline after 1 month (*n* = 10)	842 ± 85	12.1 ± 1.7	461 ± 44	93 ± 11	26 ± 4.8
	754 ± 61	10.3 ± 1.6	413 ± 42	67 ± 9	30 ± 5.1
	*P* > 0.05	*P* > 0.05	*P* > 0.05	*P* < 0.05*	*P* > 0.05

WPL baseline after 1 month (*n* = 10)	725 ± 63	15.3 ± 2.2	410 ± 61	88 ± 9	23 ± 5.6
	342 ± 27	7.8 ± 1.5	295 ± 45	48 ± 5	38 ± 5.2
	*P* < 0.001*	*P* < 0.05*	*P* < 0.05*	*P* < 0.01*	*P* < 0.01*

Placebo baseline after 1 month (*n* = 10)	720 ± 81	7.2 ± 3.1	470 ± 43	86 ± 10	25 ± 4.4
	759 ± 79	7.4 ± 3.9	495 ± 61	85 ± 12	27 ± 4.1
	*P* > 0.05	*P* > 0.05	*P* > 0.05	*P* > 0.05	*P* > 0.05

**Table 4:** Effect on cardiovascular parameters.

Product	Pulse rate per min	ABI	FMD (%)	Blood pressure, in mm Hg		StO_2_, % O_2_/min
				Systolic	Diastolic	
WP baseline after 1 month (*n* = 10)	71.0 ± 3.9	1.05 ± 0.04	9.4 ± 1.1	137 ± 9.7	86.1 ± 5.0	10.2 ± 0.9
	70.6 ± 2.8	1.06 ± 0.03	9.5 ± 1.2	135 ± 8.8	83.6 ± 3.2	10.3 ± 1.1
	*P* > 0.05	*P* > 0.05	*P* > 0.05	*P* > 0.05	*P* > 0.05	*P* > 0.05

Lycopene baseline after 1 month (*n* = 10)	73.1 ± 3.8	1.09 ± 0.05	10.5 ± 1.1	132 ± 3.6	86 ± 3.4	13.1 ± 0.8
	71.5 ± 4.9	1.01 ± 0.07	11.1 ± 0.9	130 ± 6.5	83 ± 4.8	14.7 ± 0.9
	*P* > 0.05	*P* > 0.05	*P* > 0.05	*P* > 0.05	*P* > 0.05	*P* > 0.05

WPL baseline after 1 month (*n* = 10)	68.3 ± 3.2	0.97 ± 0.07	10.3 ± 0.8	131 ± 8.0	81 ± 5.0	11.4 ± 0.6
	67.9 ± 2.9	0.97 ± 0.08	12.9 ± 0.8	124 ± 7.4	77 ± 4.1	16.4 ± 0.8
	*P* > 0.05	*P* > 0.05	*P* < 0.01*	*P* < 0.01*	*P* < 0.01*	*P* < 0.01*

Placebo baseline after 1 month (*n* = 10)	65.2 ± 5.1	0.97 ± 0.07	10.2 ± 0.9	135 ± 8.5	84 ± 4.1	11.5 ± 0.7
	66.4 ± 4.6	0.97 ± 0.08	10.0 ± 1.2	136 ± 9.6	83.5 ± 3.3	12.2 ± 0.4
	*P* > 0.05	*P* > 0.05	*P* > 0.05	*P* > 0.05	*P* > 0.05	*P* > 0.05
